# Integrating oral health into Universal Health Coverage in Europe: A cross-sectional ecological analysis of services and outcomes

**DOI:** 10.1097/MD.0000000000048072

**Published:** 2026-03-20

**Authors:** Omer Faruk Sonmez, Muhammed Ikbal Sonmez

**Affiliations:** a Division of Population Health, School of Medicine and Population Health, University of Sheffield, Sheffield, UK; b Department of Oral Health, Vocational School of Health Services, Kirikkale University, Kirikkale, Turkiye; c Department of Law, Faculty of Law, Cankiri Karatekin University, Cankiri, Turkiye.

**Keywords:** dental care access, Europe, health equity, oral health, public dental expenditure, Universal Health Coverage, unmet dental needs

## Abstract

Integration of oral health within Universal Health Coverage (UHC) remains uneven across Europe, and comparative evidence linking statutory dental coverage and public financing with oral health outcomes is limited. This study assessed relationships between integration of essential oral health services within UHC and prevalence of untreated dental caries and self-reported unmet need for dental care across European countries. A cross-sectional ecological analysis was conducted using publicly available, country-level data for 34 WHO European Region countries (indicator years 2019–2023). Outcomes were untreated dental caries prevalence (≥5 years) and self-reported unmet dental need (≥16 years). Explanatory variables were public per capita dental expenditure (United States dollars), dentists per 10,000 population, statutory dental coverage model (comprehensive/partial = reference; limited = contrast), and public share of total dental spending (%). Descriptive statistics summarized health system indicators; 2 multiple linear regression models estimated associations. Across 34 countries, median unmet need was 1.58% (interquartile range 0.56–3.97%) and untreated caries 35.41% (interquartile range 31.61–38.15%). Health system inputs varied widely: median per capita dental expenditure = USD 97.8 (10–493.7); dentist density = 7.9 per 10,000 (1.1–17.7); public share = 32.8% (0.3–72.4). Model 1 (untreated caries): *R*^2^ = 0.371; *F*(4, 29) = 4.27; *P* = .008. Higher public per capita dental expenditure was associated with lower caries prevalence (*B* = −0.045; 95% confidence interval [CI]: −0.074 to −0.015; *P* = .004). Limited statutory coverage (vs comprehensive/partial) was associated with higher caries (*B* = 6.505; 95% CI: 2.526–10.483; *P* = .002). A higher public share of dental spending correlated positively with caries prevalence (*B* = 0.210; 95% CI: 0.095–0.326; *P* < .001). Dentist density showed no association (*P* = .705). Model 2 (unmet dental need): *R*^2^ = 0.370; *F*(4, 29) = 4.27; *P* = .007. Limited statutory coverage (vs comprehensive/partial) was associated with higher unmet need (*B* = 5.735; 95% CI: 2.396–9.075; *P* = .001). Other predictors were not significant (all *P* > .35). Broader statutory dental coverage was associated with lower unmet dental need, and greater public per capita dental expenditure with lower untreated caries prevalence. Workforce density alone was not predictive. Expanding prevention-oriented essential dental benefits and strengthening financial protection are central to achieving equitable oral health within UHC.

## 1. Introduction

Universal Health Coverage (UHC), defined as ensuring all individuals have access to essential health services without financial hardship, has emerged as a critical goal in global health policy, particularly as a cornerstone of the Sustainable Development Goals by 2030.^[1]^ Despite the global momentum towards achieving UHC, oral health remains inadequately addressed within this framework, often perceived as secondary to other health priorities.^[2]^ This omission is striking, given that oral diseases are among the most common noncommunicable diseases (NCDs), affecting around 3.5 billion people worldwide and contributing significantly to overall healthcare costs and socioeconomic disparities.^[3]^

Oral health directly influences general health, affecting essential functions such as eating, speaking, and socializing, thereby impacting quality of life.^[4]^ However, in many countries, essential dental care is only partially covered, or it is entirely absent from publicly funded health services, leaving individuals to shoulder substantial out-of-pocket expenses for basic oral healthcare.^[5]^ This financial burden leads to inequalities in access to dental services and increased unmet needs, particularly among vulnerable populations, which is contrary to the core objectives of UHC which is essentially about promoting equity and financial protection.^[6]^

In the World Health Organization (WHO) European Region, more than half of adults had at least 1 major oral disease in 2019 which is the highest prevalence recorded globally, underscoring the scale of unmet oral health needs in this region.^[7]^ Untreated dental caries remains the most prevalent condition across the life course, while periodontal diseases and tooth loss contribute substantially to disability and costs.^[8]^ Marked within-country inequalities persist: recent European Union Statistics on Income and Living Conditions (EU-SILC) data show that people at risk of poverty report unmet dental care needs far more often than those not at risk (13.7% vs 5.1%), with country levels ranging from 0.4% to 27.1%.^[9]^ The disparities mirror the strong social gradient in oral diseases described by WHO, with higher burdens among groups with lower income and education and in populations facing access barriers.

The UHC framework is built upon 3 interconnected pillars: accessibility, affordability, and quality. Accessibility ensures that health services are available where and when they are needed, affordability protects individuals from financial hardship associated with healthcare costs, and quality ensures that the services provided are effective and safe^[10]^. However, it is important to clarify what UHC does not mean. UHC is not synonymous with free coverage for every possible health intervention, as no country can sustainably provide all services at no cost.^[11]^ UHC is also not limited to health financing but encompasses all aspects of the health system, such as service delivery, workforce, technology, and quality assurance.^[12]^

In most systems, inclusion of services in the essential benefits package depends on health technology assessment comparing an intervention’s incremental cost-effectiveness ratio with a country-specific threshold, alongside budget impact and equity.^[13]^ For oral health, cost-effectiveness can be systematically undervalued because generic utility instruments used to derive quality-adjusted life years (e.g., EQ-5D/SF-6D) capture pain, mastication, speech, aesthetics, and social functioning only imperfectly, may be insensitive to short episodes or recurrent conditions, and are rarely validated for children, leading to conservative utility gains compared with oral health-specific measures.^[14]^ Moreover, mapping from instruments like OHIP-14 to utilities is inconsistent, and adaptation/recall biases can further depress estimated benefits. Incremental cost-effectiveness ratio thresholds vary widely; some countries apply explicit thresholds, others use severity- or burden-adjusted ranges, and many operate with implicit or no formal thresholds so the same dental intervention may meet coverage criteria in 1 setting but not another.^[15]^

As more countries aim to incorporate UHC into their health systems, the integration of oral health is essential for achieving a truly comprehensive approach to healthcare.^[16]^ Recognizing oral health as integral to the UHC agenda can reduce healthcare disparities, improve public health outcomes, and promote social equity.^[10]^ Bernabé et al demonstrated that household spending on dental care can consume a substantial portion of disposable income, potentially pushing families below the poverty line. Policymakers should therefore prioritize the inclusion of dental care within UHC and advocate for its incorporation into health insurance packages.^[17]^ The WHO Bangkok Declaration on Oral Health named “No Health Without Oral Health” that was adopted in Bangkok, Thailand, on 29 November 2024, highlights the urgent need to address persistent unmet oral health needs and their disproportionate burden among vulnerable populations, emphasizing integration of essential oral health services into UHC by 2030. This global call to action underscores the importance of equitable access, prevention, and early intervention to reduce the social and economic impacts of oral diseases.^[18,19]^

Europe offers a unique context for examining the integration of oral health within UHC. European countries display a wide variety of health system models and financing structures, from comprehensive national health systems to mixed public-private arrangements.^[20]^ Countries have limited, partial, comprehensive coverage on essential services. Essential oral health service coverage across European nations can offer insights into the specific policy and structural challenges that need to be addressed. This paper examines the current landscape of oral health within UHC frameworks across Europe.

This study was motivated by the recognition that, despite growing international commitments to UHC, oral health services remain fragmented and inconsistently covered across Europe. Our aim was to assess, across 34 countries in the WHO European Region, the relationship between the integration of essential oral health services within UHC and the prevalence of untreated dental caries in persons aged ≥5 years and self-reported unmet need for dental examinations among adults aged ≥16 years (indicator years 2019–2023).

## 2. Materials and methods

### 2.1. Study design and period

This was a cross-sectional ecological analysis using publicly available, country-level secondary data. Indicator years were the latest available for each source: untreated caries (2019), dentist density (2019), public per capita dental expenditure (2021), public share of total dental spending (2021), and self-reported unmet dental need (2022–2023; nearest year used where necessary). All datasets were accessed on 18 October 2024.

### 2.2. Study population/setting & sample size

The analysis covered 34 countries from the WHO European Region (not limited to EU member states), contingent on data availability. Countries with missing data for any variable in a given model were excluded listwise for that model. The full country list is provided in Table S1, Supplemental Digital Content, https://links.lww.com/MD/R551; final analytic Ns for each model are reported with the regression results.

### 2.3. Study procedures/data collection

Two primary outcomes were analyzed: the prevalence of untreated caries in individuals aged 5 and above (%), and self-reported unmet needs for dental examinations due to cost, waiting times, or travel distance for both genders age groups 16 and older (%). Independent variables included public per capita dental expenditure (in US dollars), the number of dentists per 10,000 population, the category of dental coverage (1 = comprehensive/partial or 2 = limited), and the percentage of public spending (government and compulsory insurance schemes) out of total dental care spending. Each of these variables reflects different dimensions of healthcare access, financial protection, and system capacity.

### 2.4. Study variables/outcomes

Two key dependent variables are selected to measure access to and outcomes of oral healthcare across Europe, providing insight into both service accessibility and population health status:

Self-reported unmet needs for dental examination (%): this variable retrieved from Eurostat and reflects the percentage of individuals who report unmet needs for dental examinations due to cost, waiting times, or travel distance representing access to dental care, highlighting potential barriers related to affordability, service availability, and geographical accessibility.^[21]^ For the regression analysis, the self-reported unmet dental needs indicator was recalculated as an age-standardized rate using Eurostat’s age-group breakdowns, ensuring comparability across countries with different population age structures.

Prevalence of untreated caries of permanent teeth in people aged 5 and above (%): this indicator measures the percentage of people with untreated caries capturing the extent of unmet clinical needs retrieved from WHO global health observatory.^[22]^ Estimated prevalences from the Institute of Health Metrics and Evaluation Global Burden of Disease Study 2019 (GBD2019) were computed by using population data from United Nations Department of Economic and Social Affairs (UN DESA) World Population Prospects 2019 and applying them to disease relevant age groups. High prevalence of untreated caries indicates limited access to preventive and treatment services, suggesting gaps in service delivery or financial protection mechanisms within the UHC framework.

The independent/controlling variables in both regression models represent structural and financial components of oral healthcare within UHC. Each is theoretically relevant to the study’s objective of evaluating oral health integration within UHC:

Annual national per capita expenditure on outpatient dental care (US Dollars; public and private): this variable was retrieved from Organization for Economic Co-operation and Development (OECD) health indicators.^[23]^ It reflects the financial resources dedicated to dental care in each country. This variable is instrumental in assessing whether countries with higher expenditure experience lower unmet needs and reduced prevalence of untreated oral conditions.

Number of dentists per 10,000 population: the availability of dental professionals is a crucial indicator of healthcare accessibility, and it retrieved from WHO global health observatory.^[24]^ A higher density of dentists may contribute to improved service accessibility, reduced waiting times, and increased service quality.

Category of coverage of statutory dental care (1 = comprehensive/partial or 2 = limited): this categorical variable classifies countries based on the extent of essential dental care coverage within their statutory health systems. The categories are partial and comprehensive coverage or limited coverage and retrieved from the oral health report of European Observatory on Health Systems and Policies^[20]^ for most countries. For other countries a literature search carried out to assign to one of the coverage models. UHC aims to expand coverage and financial protection, so countries with more comprehensive essential dental coverage are expected to exhibit lower rates of unmet needs and untreated caries, indicating better integration of oral health into UHC.

Percentage of public spending on dental care out of total spending (%): this variable is retrieved from OECD health indicators and indicates the share of public funding (government and compulsory schemes) in total dental care expenditure (government as well as voluntary schemes and out-of-pocket payments), reflecting the extent of financial protection offered by the state.^[25]^ There is no sustainable way of 100% oral health service coverage therefore the results only discuss inclusion of essential oral health services UHC. The data analyzed represents coverage for basic and necessary dental services, such as preventive care and treatment of common conditions like caries, extractions rather than comprehensive coverage for all dental interventions such as dental implants.

### 2.5. Data analysis

Data distribution was assessed using the Shapiro–Wilk test; means (standard deviation) were used for normally distributed variables and medians (interquartile range) for skewed variables. To assess the integration of oral health into the UHC framework across 34 European countries, a multiple linear regression analysis was conducted. This analysis is designed to explore the relationship between key indicators of oral health access and coverage within the context of UHC.^[26]^ The study employs 2 separate regression models with distinct dependent variables and a common set of independent variables to capture a comprehensive picture of oral health integration in these countries.^[27]^

For descriptive contrasts (Table S2, Panel B, Supplemental Digital Content, https://links.lww.com/MD/R551), countries were ranked separately for each system characteristic and divided into quartiles using a rank-based equal-count approach (N = 34; quartiles contained 8–9 countries), with the “Range” column reporting the minimum–maximum value within each quartile.

Collinearity diagnostics were conducted to ensure no multicollinearity issues among predictors. Collinearity was assessed using pairwise correlation matrices (Figs. S1 and S2, Supplemental Digital Content, https://links.lww.com/MD/R551) and variance inflation factors (VIF) with tolerance statistics. Correlation matrices were used to visually screen the correlation structure among predictors, while VIF/tolerance values were used to confirm that multicollinearity was not problematic for model estimation. Statistical analyses were performed in IBM SPSS Statistics for Windows, version 29.0.1.1 (IBM Corp., Armonk), with a significance level set at α = 0.05.

### 2.6. IRB oversight

IRB oversight was not applicable. The study used publicly available, aggregated secondary data with no human participants or identifiable information; ethics/IRB review and informed consent were not required under prevailing regulations.

## 3. Results

A total of 34 countries in the WHO European Region were included in the final analysis. Table S1, Supplemental Digital Content, https://links.lww.com/MD/R551, provides the country list and country-level values for, in column order: total dental expenditure in million, per capita dental expenditure in United States dollars, statutory dental coverage model, public share of total dental spending, dentist density per 10,000, self-reported unmet dental need, and prevalence of untreated caries. Table 1 summarizes descriptive statistics for the study outcomes and health system characteristics, including self-reported unmet dental need, untreated caries prevalence, per capita dental expenditure, dentist density, and the public share of total dental spending.

**Table 1:** Oral health outcomes and health system characteristics among 34 countries in the WHO European Region (indicator years 2019–2023): descriptive statistics for self-reported unmet dental need, untreated caries prevalence, per capita dental expenditure, dentist density, and public share of dental spending.

Variable	Mean (SD)	Median [IQR]	Min–Max
Self-reported unmet need (%)	–	1.58 [0.56–3.97]	0.14–14.59
Untreated caries prevalence (%)	34.72 (4.48)	–	24.56–41.31
Per capita dental expenditure (USD)	–	97.80 [47.72–230.68]	10.00–493.70
Dentist density (per 10,000)	8.33 (3.68)	–	1.10–17.70
Public share of dental spending (%)	30.82 (19.59)	–	0.30–72.40
Statutory dental coverage model, n (%)	Comprehensive/partial (reference): 24 (70.6%); Limited: 10 (29.4%)		

Mean (SD) is presented for normally distributed variables; median [IQR] is presented for skewed variables (distribution assessed using Shapiro–Wilk test). Coverage model coded 1 = comprehensive/partial (reference); 2 = limited.

IQR = interquartile range, SD = standard deviation, USD = United States dollars.

### 3.1. Health system characteristics and oral health outcomes

Across 34 countries, self-reported unmet dental need was generally low (median 1.58%) but highly heterogeneous (0.14–14.59%). Untreated caries remained substantial (median 35.41%; interquartile range 31.61–38.15%). Health system inputs varied widely: median per capita dental spending was $97.80 (range $10.00–$493.70), dentist density 7.90 per 10,000 (1.10–17.70), and the public share of dental spending 32.80% (0.30–72.40), indicating large cross-country differences in financial protection can be seen in Table 1 below.

### 3.2. Relationship between health system characteristics and oral health outcomes

Descriptive contrasts in oral health outcomes by statutory coverage model and by quartiles of system characteristics are presented in Table S2, Supplemental Digital Content, https://links.lww.com/MD/R551. Countries with limited statutory dental coverage had higher median untreated caries prevalence (36.8% vs 33.9%) and substantially higher median unmet dental need (7.66% vs 1.27%) compared with countries with comprehensive/partial coverage (Table S2, Panel A, Supplemental Digital Content, https://links.lww.com/MD/R551). Across quartiles of per capita dental expenditure, untreated caries showed a downward gradient from the lowest to the highest quartile (median 37.86% in Q1 vs 32.5% in Q4), whereas unmet need showed no consistent monotonic pattern across expenditure quartiles (Table S2, Panel B, Supplemental Digital Content, https://links.lww.com/MD/R551). In contrast, dentist density showed no clear gradient for either outcome across quartiles. A higher public share of dental spending was associated with lower unmet need (median 3.32% in the lowest quartile vs 0.56% in the highest quartile), but caries prevalence did not decrease consistently across public share quartiles (Table S2, Panel B, Supplemental Digital Content, https://links.lww.com/MD/R551).

### 3.3. Determinants of untreated caries

The multiple linear regression analysis examining the prevalence of untreated caries across 34 European countries explained 37.1% of the variance in the outcome as seen Table 2 below, indicating that the model was statistically significant.

**Table 2:** Determinants of untreated dental caries (≥5 years) in 34 countries in the WHO European Region (indicator years 2019–2023): multivariable linear regression of caries prevalence on UHC integration indicators (per capita expenditure, dentist density, statutory coverage model, and public share of dental spending).

Variable	B (unstandardized coefficients)	95% CI for B	β	*t*	*P*-value	Tolerance	VIF
Constant	22.583	(15.427, 29.740)	N/A	6.454	.001	N/A	N/A
Public per capita dental expenditure	−0.045	(−0.074, −0.015)	−0.614	−3.084	.004	0.547	1.827
Dentists per 10k population	−0.00007	(−0.000, 0.000)	−0.059	−0.382	.705	0.909	1.101
Dental coverage model*	6.505	(2.526, 10.483)	0.672	3.344	.002	0.538	1.860
Percentage of public spending on dental care	0.210	(0.095, 0.326)	−0.920	−3.728	<.001	0.356	2.806

CI = confidence interval, UHC = Universal Health Coverage, VIF = variance inflation factor, WHO = World Health Organization.

* Coverage model coded 1 = comprehensive/partial (reference); 2 = limited.

The model was significant (*R*^2^ = 0.371; *F*(4,29) = 4.27; *P* = .008). Public per capita dental expenditure was significantly negatively associated with the prevalence of untreated caries (*B* = –0.045, *P* = .004). Dental coverage model was significantly positively associated with untreated caries prevalence (*B* = 6.505, *P* = .002). Percentage of public spending on dental care was positively associated with untreated caries prevalence (*B* = 0.210, *P* < .001). Dentists per 10,000 population was not significantly associated with the prevalence of untreated caries (*B* = –0.00007, *P* = .705).

### 3.4. Determinants of unmet need for dental care

The regression model for self-reported unmet dental needs accounted for 37% of the variance in the outcome also indicating statistical significance as seen Table 3 below.

**Table 3:** Determinants of self-reported unmet need for dental examinations (≥16 years) in 34 countries in the WHO European Region (indicator years 2019–2023): multivariable linear regression on UHC integration indicators (per capita expenditure, dentist density, statutory coverage model, and public share of dental spending).

Variable	B (unstandardized coefficients)	95% CI for B	β	*t*	*P*-value	Tolerance	VIF
Constant	1.289	(−1.871, 4.448)	N/A	0.834	.411	N/A	N/A
Public per capita dental expenditure	−0.004	(−0.013, 0.005)	−0.151	−0.916	.367	0.696	1.436
Dentists per 10k population	0	(0, 0)	0.055	0.37	.714	0.873	1.145
Dental coverage model*	5.735	(2.396, 9.075)	0.735	3.513	.001	N/A	N/A
Percentage of public spending on dental care	0.013	(−0.059, 0.086)	0.072	0.374	.711	0.511	1.956

CI = confidence interval, UHC = Universal Health Coverage, VIF = variance inflation factor, WHO = World Health Organization.

* Coverage model coded 1 = comprehensive/partial (reference); 2 = limited.

The model was significant (*R*^2^ = 0.370; *F*(4,29) = 4.27; *P* = .007). Dental coverage model was the only variable significantly associated with self-reported unmet dental needs (*B* = 5.735, *P* = .001). Public per capita dental expenditure was not significantly associated with unmet needs (*B* = –0.004, *P* = .367).

Dentists per 10,000 population was not significantly associated with unmet needs (*B* = 0.000, *P* = .714). Percentage of public spending on dental care was not significantly associated with unmet needs (*B* = 0.013, *P* = .711).

Pairwise correlations between independent variables are presented as figures in Figure S1, Supplemental Digital Content, https://links.lww.com/MD/R551 represents correlation matrix for caries prevalence model and Figure S2, Supplemental Digital Content, https://links.lww.com/MD/R551, represents correlation matrix for unmet dental need. These figures were used as part of the collinearity assessment and are consistent with the VIF/tolerance diagnostics reported in Tables 2 and 3, supporting retention of the prespecified predictors in the multivariable models.

## 4. Discussion

This study assessed, across 34 countries in the WHO European Region, how integration of essential oral health services within UHC relates to prevalence of untreated dental caries (≥5 years) and self-reported unmet need for dental examinations (≥16 years). Four findings emerged.

Higher public per capita expenditure on essential dental care was associated with lower prevalence of untreated caries, supporting the view that increased investment in dental services can contribute to reducing the burden of untreated disease. In contrast, countries with more limited or no essential dental coverage reported higher levels of both untreated caries and unmet dental needs, highlighting the importance of financial protection in improving access to care.

The observed positive association between the percentage of public spending within overall dental expenditure and untreated caries prevalence is counterintuitive. One possible explanation is that higher public investment may coincide with stronger access, leading to greater detection and recording of untreated caries.

As oral health is gaining recognition as a core component of UHC due to its direct impact on overall health and its status as the number 1 NCD, integrating essential oral health care into UHC is crucial for promoting equity in health.

### 4.1. Increase coverage on essential oral health services

Among the 34 WHO European Region countries in our sample, 2 broad statutory coverage models were observed. Most operated a “comprehensive/partial” benefits model, offering publicly financed essential services that are typically periodic examinations, prevention (e.g., prophylaxis/fluoride), basic restorations and extractions often with co-payments, caps, or narrower adult benefits; orthodontics and advanced prosthodontics were commonly excluded or tightly rationed. The remainder (29%) had “limited” coverage focused on urgent care and/or targeted groups (e.g., children, low-income, medically vulnerable), with routine adult care predominantly paid out-of-pocket. Financing arrangements spanned tax-funded national health systems and social health insurance schemes, but the functional scope of covered dental benefits, rather than the financing label, appeared most salient for access and outcomes in our analysis.

Consistent with our hypothesis, higher public per capita dental expenditure was independently associated with lower prevalence of untreated caries (Table 2). This suggests that greater government investment in essential dental services can reduce untreated disease, in line with prior work.^[20,28]^ In contrast, the public share of total dental spending was positively associated with caries. This likely reflects denominator and context effects, for example small overall dental budgets or a larger underlying disease burden where governments shoulder a higher share, and policy endogeneity. Overall, the absolute level and preventive orientation of public spending appear more consequential than the public fraction of a small total budget.

The results show that higher public per capita expenditure on dental care in Europe may reduce the prevalence of untreated caries. Oral diseases remain prevalent despite being preventable, particularly due to limited essential public coverage and reliance on out-of-pocket payments. On average, only one-third of dental care spending is publicly funded, leaving many individuals unable to afford care.^[17,28]^ Expanding public financial protection is essential for ensuring access to basic dental services and reducing untreated oral conditions.

The positive association between the share of public spending in total dental care and untreated caries prevalence may arise from policy gaps in dental coverage – countries with limited public funding often see individuals delaying care until it becomes urgent, especially among low-income groups^[29,30]^

Similarly, the social importance of dental aesthetics may drive individuals to prioritize aesthetic or cosmetic treatments out-of-pocket even in low-coverage systems, a tendency linked to social acceptance and quality of life.^[31]^ This dynamic can render such expenditures inelastic while public funding remains focused on acute or complex interventions, which may in turn drive up recorded prevalence of untreated caries. This likely reflects budgets favoring complex treatment and private spending on aesthetics, underfunding prevention; prioritizing preventive and essential services is warranted.

### 4.2. Improve accessibility and innovate dental care models

Although dentist density was not a significant predictor of reduced caries prevalence or unmet needs in this study, the report highlights geographical disparities in the distribution of dental professionals, particularly in rural and underserved areas. Many European countries face challenges in providing equitable access to dental care, especially where dentist shortages are more pronounced in rural regions.^[32]^ Policymakers should address these geographic inequities by ensuring an adequate distribution of dental professionals, expanding public dental services in rural areas, and reducing travel distances for patients.

To improve access within UHC frameworks, innovative care models are essential. Prasad et al has demonstrated, 1 effective approach as enhancing care coordination, where medical providers deliver basic preventive oral care and refer patients to dental professionals for more complex treatments, ensuring early intervention for vulnerable populations as well as co-locating dental hygienists in primary care settings. Telehealth models also offer solutions to geographic barriers by allowing dental hygienists to provide care in remote areas, with consultations from dentists conducted virtually. Lastly, interprofessional collaboration, where healthcare providers from different disciplines work together, can enhance comprehensive care and improve early detection of oral health issues. Expanding the role of dental hygienists and promoting interprofessional models will be key to integrating oral health into UHC and improving outcomes.^[33]^

Across countries, dentist density varied but was not associated with untreated caries or unmet need in adjusted models. This suggests national headcounts are a weak proxy for access and outcomes relative to coverage design and financial protection. We do not infer within-country patterns from these data; assessing maldistribution and skill mix will require subnational metrics beyond this study’s scope.

### 4.3. Broaden coverage beyond treatment

The results found that broader dental coverage was associated with decreased unmet needs. Unmet dental care needs remain the highest compared to other healthcare needs, particularly among low-income populations.^[34]^ However, countries with higher public funding for dental care may still experience significant burdens of untreated caries, possibly due to inefficiencies in resource allocation or a larger preexisting burden of oral health issues. It may also indicate that higher public spending is not always directed toward preventive care but is instead focused on managing existing oral health problems. To address this, UHC frameworks must go beyond coverage for treatment and include preventive care, such as oral health education, regular checkups, and fluoride treatments. Preventive care reduces the long-term burden of oral diseases and is a cost-effective strategy that promotes better overall health outcomes.

It is plausible that broader statutory dental coverage may reduce unmet need more quickly than it reduces untreated caries prevalence, because caries is cumulative and reflects long-run exposures (e.g., diet, fluoride environment) and access barriers accumulated over years; accordingly, population-level prevalence may change more slowly and may not track short-term policy changes. Importantly, with this cross-sectional ecological design and non-contemporaneous indicator years, we cannot isolate the independent effect of “duration of coverage” from other factors such as baseline disease burden, prevention intensity, service utilization patterns, or broader social determinants. An illustrative example from our analytic sample is Türkiye, which we categorized as comprehensive/partial statutory dental coverage yet showed a high prevalence of untreated caries in 2019 (40.31%). While Türkiye’s Health Transformation Program (2003) marked a major expansion of the public role and capacity in service delivery, the continued characterization of the system as largely treatment-oriented and the inconsistent availability of preventive services suggest that coverage breadth alone may be insufficient to reduce disease burden unless accompanied by sustained, prevention-oriented implementation. This example therefore highlights that observed cross-country differences in untreated caries may reflect a combination of benefit design, the prevention-treatment balance, implementation fidelity, and contextual determinants over time, rather than duration of coverage in isolation.^[35]^

### 4.4. Integrate oral health into broader health policies

The results emphasize the importance of integrating oral health with other public health strategies, particularly given the shared risk factors between oral diseases and other NCDs, such as smoking, poor diet, and alcohol use. The inclusion of oral health in UHC can help address these common risk factors through a holistic approach to healthcare. Policymakers should promote the integration of oral health into broader health policies, ensuring that oral health promotion and prevention are prioritized alongside general healthcare initiatives.

To strengthen equity in oral health integration into UHC, governments can target subsidies for vulnerable groups, embed basic dental services in primary care, and link public funding to measurable improvements in access and outcomes. Partnerships between health, education, and social sectors can also address upstream determinants, reducing inequities over time.

### 4.5. Strengths

This study has several strengths. First, it provides a multi-country comparative assessment of oral health integration within UHC across 34 countries in the WHO European Region, capturing substantial heterogeneity in financing, coverage design, and workforce capacity and enabling cross-national inference within the limits of ecological data. Second, we used publicly available, internationally recognized data sources (e.g., WHO/GBD, OECD, Eurostat) and harmonized indicators to maximize comparability across settings and enhance transparency and reproducibility. Third, the analytic approach incorporated prespecified multivariable models aligned with the study aim and included collinearity diagnostics (correlation matrices and VIF/tolerance), supporting the stability and interpretability of coefficient estimates. Fourth, we complemented regression findings with structured descriptive contrasts (Table S2, Supplemental Digital Content, https://links.lww.com/MD/R551) to improve interpretability of cross-country patterns. Finally, the direction of several findings – particularly the association of broader statutory coverage with lower unmet need and the inverse association of per capita public expenditure with untreated caries – is consistent with the conceptual framework that financial protection and investment are central mechanisms through which UHC may improve access and outcomes, supporting the credibility of the observed relationships.

### 4.6. Limitations

The analysis includes 34 European countries as presented in Supplementary Digital Content, resulting in a relatively small sample size for the number of explanatory variables used in the multiple regression models. This raises concerns about overfitting, limits statistical power, and restricts the robustness of the findings. Larger datasets or longitudinal panel data would be more appropriate for exploring complex relationships between oral health and healthcare system indicators.

Because the study is cross-sectional and indicator years are not fully aligned (caries 2019 vs spending 2021), the inverse association between per capita public expenditure and caries is not causal. Spending may respond to higher disease burden and unmeasured confounding may influence both variables. Per capita public outlays and the public share capture different constructs, so their divergence may reflect denominator effects and policy responses.

Some of the explanatory variables used may be correlated with one another. For example, public per capita dental expenditure and the percentage of public spending on dental care both represent different aspects of financing but may be conceptually and statistically interrelated. Although collinearity diagnostics were performed, the potential overlap in capturing public investment may distort the unique contribution of each variable.

Not all variables were obtained from the same year, which may compromise the internal validity of the associations. For instance, untreated caries data were sourced from 2019, while expenditure and workforce indicators were from later years (e.g., 2021), introducing temporal misalignment. As such, correlations between inputs and outcomes should be interpreted with caution.

The outcome variable of self-reported unmet dental needs is subject to recall and perception bias, which may differ substantially between countries due to variations in survey methodology, healthcare expectations, and cultural norms. Additionally, this indicator was not age-standardized, despite known differences in oral healthcare needs across age groups – particularly for older adults.

The binary classification of dental coverage (e.g., limited vs partial/comprehensive) may oversimplify nuanced national policies and mask variability within each group. Differences in eligibility, scope of services, and implementation across countries that fall under the same category may reduce the precision of the model. A more granular or ordinal classification could better reflect policy differences.

The study does not account for several important contextual or structural factors that likely influence oral health outcomes. These include income inequality, education levels, diet, sugar consumption, fluoride exposure, and health literacy. Regional disparities in dentist distribution, urban–rural differences, and broader health system performance (e.g., efficiency, integration, or care navigation) were also not included. These unmeasured confounders may have influenced both access and outcomes, weakening the explanatory capacity of the current model.

Although higher public per capita spending was associated with reduced caries prevalence, the positive association found between the percentage of public spending and caries prevalence suggests more complex dynamics. It may reflect unmet demand, greater disease burden, or inefficiencies in how funds are allocated (e.g., reactive treatment rather than prevention). These findings highlight the limitation of interpreting spending as a direct proxy for service effectiveness or equity. Although the self-reported unmet dental needs indicator is collected for both sexes aged 16 and older, this study did not conduct a gender-disaggregated analysis. As such, potential gender differences in access or outcomes were not examined and may represent an unmeasured source of variation. Future research could explore sex- and gender-specific patterns in unmet dental needs to inform more targeted policy interventions.

In interpreting the results, our analytic sample comprised 34 WHO European Region countries selected on data availability, predominantly high-income, so generalisability is strongest to similar settings; listwise exclusion may introduce selection bias, though substantial heterogeneity in financing, coverage and workforce remains (Table S1, Supplemental Digital Content, https://links.lww.com/MD/R551). Key system characteristics varied widely (Table 1; Table S2, Supplemental Digital Content, https://links.lww.com/MD/R551), aiding identification of associations but also capturing unmeasured factors (e.g., prevention orientation, diet/fluoride exposure, oral health literacy) that may influence estimates. Consistent with regional reports, untreated caries remained common with notable cross-country variation, indicating that overall spending alone is insufficient to reduce disease. In multivariable analyses (Table 2), higher public per capita dental expenditure was associated with lower caries prevalence, whereas limited statutory coverage (vs comprehensive/partial) aligned with higher prevalence; the positive association between the public share of dental spending and caries may reflect higher underlying need, detection effects, or spending weighted toward treatment rather than prevention. Dentist density showed no association, suggesting that how care is financed and organized, particularly the breadth of prevention-oriented benefits; may be more consequential for outcomes than headcount alone.

## 5. Conclusions

Integrating oral health into UHC frameworks may be an important strategy for promoting health equity. In this multi-country analysis, broader statutory coverage of essential dental services was associated with lower unmet need, and higher public per capita dental expenditure with lower prevalence of untreated caries. Dentist density showed no association with either outcome, indicating that workforce numbers alone are insufficient without financial protection and service design that enable access. A higher public share of total dental spending correlated with higher caries prevalence, likely reflecting context (legacy burden and allocation toward treatment rather than prevention) rather than the level of spending per se. Substantial cross-country heterogeneity underscores the importance of system design and benefit scope.

In practice, health systems should expand prevention-oriented essential dental benefits within UHC, strengthen financial protection, especially for low-income and remote populations, and tie public outlays to measurable indicators of access and prevention while monitoring equity. For research, priorities are to use longitudinal or quasi-experimental designs, apply more granular classifications of coverage, include sex-disaggregated analyses when data permit, and improve outcome measures that capture oral health related quality of life for health technology assessment.

The chosen indicators in this study are generally well-suited to assess the degree of oral health integration within UHC in European countries, as they reflect financial commitment, service availability, and system capacity. The variables collectively capture the core dimensions of accessibility, affordability, and service outcomes as they relate to oral health. However, several important limitations must be acknowledged, which affect the interpretation and generalizability of the results.

## Author contributions

**Conceptualization:** Omer Faruk Sonmez, Muhammed Ikbal Sonmez.

**Data curation:** Omer Faruk Sonmez.

**Formal analysis:** Omer Faruk Sonmez.

**Methodology:** Omer Faruk Sonmez.

**Supervision:** Omer Faruk Sonmez.

**Writing – original draft:** Omer Faruk Sonmez.

**Writing – review & editing:** Muhammed Ikbal Sonmez.

## Supplementary Material


