# Supplementary material for: Integrating oral health into Universal Health Coverage in Europe: A cross-sectional ecological analysis of services and outcomes
Source: Medicine (Baltimore). 2026 Mar 20;105(12):e48072. doi: 10.1097/MD.0000000000048072 (PMC13008190; doi:10.1097/MD.0000000000048072)

**Supplementary Table 1. Country list and source values for outcomes and covariates for 34 countries in the WHO European Region (indicator years 2019–2023): total dental expenditure (million), per-capita dental expenditure (USD), statutory dental coverage model, public share of total dental spending (%), dentist density (per 10,000), self-reported unmet dental need (%), and prevalence of untreated caries (%).**

| **Country** | **Total dental expenditure (million USD)** | **Per-capita dental expenditure (USD)** | **Statutory dental coverage model** | **Public share of total dental spending (%)** | **Dentists per 10,000 population** | **Self-reported unmet dental need (%)** | **Prevalence of untreated caries (%)** |
| --- | --- | --- | --- | --- | --- | --- | --- |
| Albania | 36.32 | 12.7 | 2 | 18 | 10.334 | 14.589 | 39.76 |
| Austria | 2116 | 236.4 | 1 | 47.7 | 6.225 | 0.449 | 29.86 |
| Belgium | 2122 | 185.2 | 1 | 32.6 | 11.606 | 2.724 | 27.39 |
| Bulgaria | 109.4 | 15.7 | 1 | 50 | 15.609 | 1.583 | 38.18 |
| Croatia | 149.4 | 36.8 | 1 | 61.8 | 13.505 | 0.244 | 40.69 |
| Cyprus | 12.62 | 14.4 | 1 | 1 | 8.029 | 1.515 | 33.77 |
| Czechia | 843.8 | 79.3 | 1 | 43.2 | 7.576 | 0.579 | 37.3 |
| Denmark | 1868 | 321.7 | 1 | 34.9 | 7.151 | 7.551 | 24.56 |
| Estonia | 149 | 113.0 | 1 | 27.4 | 1.2 | 2.941 | 31.65 |
| Finland | 593.7 | 107.6 | 1 | 40.7 | 9.684 | 0.515 | 29.66 |
| France | 12666 | 195.4 | 1 | 67.3 | 6.955 | 5.097 | 36.82 |
| Germany | 30877 | 372.2 | 1 | 72.4 | 8.545 | 0.27 | 31.65 |
| Greece | 826.5 | 77.1 | 2 | 0.3 | 13.172 | 10.621 | 37.44 |
| Hungary | 363.9 | 37.3 | 1 | 34.4 | 7.1 | 0.519 | 37.86 |
| Iceland | 145.5 | 407.5 | 2 | 31.7 | 8.072 | 9.076 | 40.47 |
| Ireland | 514.8 | 104.0 | 1 | 44 | 4.818 | 1.153 | 31.6 |
| Italy | 1827 | 316.8 | 2 | 5 | 8.393 | 1.603 | 29.58 |
| Latvia | 19123 | 58.1 | 2 | 12.1 | 7.199 | 8.737 | 35.67 |
| Lithuania | 112 | 71.5 | 1 | 12.7 | 14.229 | 1.866 | 37.28 |
| Luxembourg | 199 | 345.4 | 1 | 47.7 | 9.743 | 0.556 | 32.5 |
| Malta | 212 | 91.6 | 1 | 30 | 6.607 | 0.187 | 33.6 |
| Netherlands | 44.45 | 213.5 | 2 | 12.4 | 5.727 | 0.19 | 38.77 |
| North Macedonia | 5.72 | 15.3 | 1 | 12 | 7.773 | 2.03 | 29.22 |
| Norway | 3678 | 405.0 | 2 | 27.1 | 9.291 | 8.31 | 39.75 |
| Poland | 31.89 | 44.8 | 1 | 35 | 9.104 | 0.831 | 35.14 |
| Portugal | 2169 | 84.7 | 2 | 14 | 11.819 | 7.013 | 29.8 |
| Romania | 1702 | 19.03 | 1 | 7 | 1.1 | 3.732 | 27.52 |
| Slovakia | 869.5 | 56.5 | 1 | 52.4 | 5.278 | 0.937 | 41.31 |
| Slovenia | 376.1 | 58.7 | 1 | 44.3 | 7.441 | 0.137 | 38.05 |
| Spain | 308.2 | 178.0 | 2 | 1.8 | 8.511 | 4.05 | 34.68 |
| Sweden | 121.5 | 327.0 | 1 | 42.9 | 17.7 | 1.568 | 34.04 |
| Switzerland | 8306 | 493.7 | 2 | 7.7 | 4.059 | 3.315 | 36.15 |
| Türkiye | 3376 | 10.0 | 1 | 33 | 4.701 | 1.458 | 40.31 |
| United Kingdom | 4219 | 143.2 | 1 | 43.3 | 4.931 | 1.397 | 38.57 |

Notes: Coverage model coded 1 = comprehensive/partial (reference); 2 = limited.

**Supplementary Table 2:** Descriptive contrasts in oral health outcomes by statutory coverage model and by quartiles of system characteristics among 34 countries in the WHO European Region (indicator years 2019–2023): Panel A—outcomes by coverage model; Panel B—outcomes by quartiles of per-capita expenditure, dentist density, and public share of spending.

Panel A. Outcomes by coverage model

| Coverage_label | Countries | Caries (median [IQR]) | Unmet need (median [IQR]) |
| --- | --- | --- | --- |
| Comprehensive/Partial (ref) | 24 | 33.9 [31.16–37.91] | 1.27 [0.52–1.91] |
| Limited | 10 | 36.8 [34.93–39.51] | 7.66 [3.50–8.99] |

Panel B. Outcomes by quartiles of system characteristics

| Characteristic | Quartile | Countries | Range | Caries (median [IQR]) | Unmet need (median [IQR]) |
| --- | --- | --- | --- | --- | --- |
| Per-capita expenditure (USD) | Q1 (lowest) | 9 | 10.0–44.8 | 37.86 [33.77–39.76] | 1.52 [0.83–2.03] |
| Per-capita expenditure (USD) | Q2 | 8 | 56.5–91.6 | 37.29 [35.15–37.59] | 1.4 [0.48–7.44] |
| Per-capita expenditure (USD) | Q3 | 8 | 104.0–213.5 | 33.16 [31.12–37.26] | 2.06 [0.99–3.22] |
| Per-capita expenditure (USD) | Q4 (highest) | 9 | 236.4–493.7 | 32.5 [29.86–36.15] | 1.6 [0.56–7.55] |
| Dentist density (per 10,000) | Q1 (lowest) | 9 | 1.1–6.22 | 36.15 [31.60–38.77] | 1.4 [0.94–2.94] |
| Dentist density (per 10,000) | Q2 | 8 | 6.61–7.77 | 36.25 [32.51–37.44] | 1.3 [0.44–5.71] |
| Dentist density (per 10,000) | Q3 | 8 | 8.03–9.68 | 34.22 [31.15–36.29] | 1.56 [0.75–5.12] |
| Dentist density (per 10,000) | Q4 (highest) | 9 | 9.74–17.7 | 37.28 [32.50–38.18] | 1.87 [1.57–7.01] |
| Public share of dental spending (%) | Q1 (lowest) | 9 | 0.3–12.4 | 34.68 [29.58–36.15] | 3.32 [1.60–4.05] |
| Public share of dental spending (%) | Q2 | 8 | 12.7–32.6 | 35.44 [31.19–39.75] | 4.98 [2.51–8.50] |
| Public share of dental spending (%) | Q3 | 8 | 33.0–43.3 | 36.22 [32.95–38.04] | 1.11 [0.56–1.49] |
| Public share of dental spending (%) | Q4 (highest) | 9 | 44.0–72.4 | 36.82 [31.65–38.18] | 0.56 [0.27–1.15] |

**Notes:** Coverage model coded 1 = comprehensive/partial (reference), 2 = limited. Quartiles were created separately for each system characteristic by ranking countries and assigning them to four approximately equal-sized groups (N=34; 9–8–8–9 countries per quartile); ranges show the observed minimum–maximum within each quartile. Values are medians [IQR] for outcomes; ranges show the quartile span of the system characteristic.

***Supplementary Figure 1.****Correlation matrix used for collinearity diagnostics in the caries model (Model 1) for 34 countries in the WHO European Region (indicator years 2019–2023).*


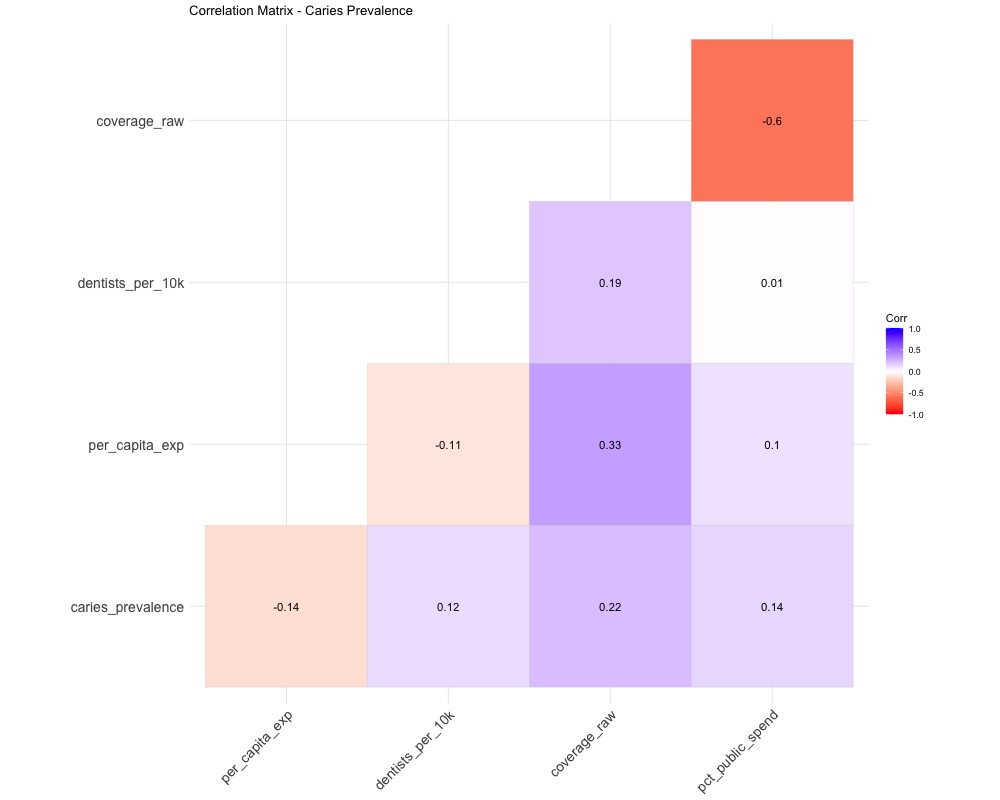


**Supplementary Figure 2 .** *Correlation matrix used for collinearity diagnostics in the unmet-need model (Model 2) for 34 countries in the WHO European Region (indicator years 2019–2023).*
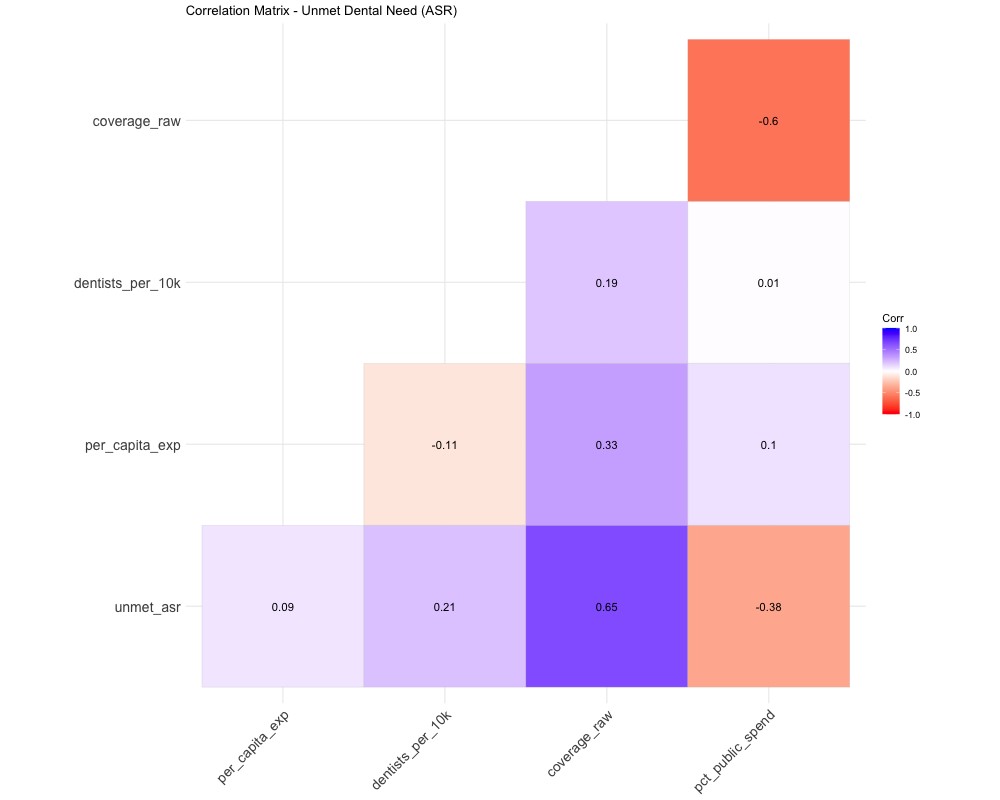

Supplement: Supplementary file 1 [file medi-105-e48072-s001.docx]
